# Hospital-based Introduction of Untested High-risk Foods for Down Syndrome Infant with Severe Food Protein-induced Enterocolitis Syndrome: A Case Report

**DOI:** 10.31662/jmaj.2024-0188

**Published:** 2024-12-06

**Authors:** Chisato Jimbo, Kouhei Hagino, Daichi Suzuki, Tomoki Yaguchi, Marei Omori, Daisuke Harama, Kotaro Umezawa, Sayaka Hamaguchi, Fumi Ishikawa, Seiko Hirai, Kenji Toyokuni, Tatsuki Fukuie, Yukihiro Ohya, Kiwako Yamamoto-Hanada

**Affiliations:** 1 Allergy Center, National Center for Child Health and Development, Tokyo, Japan

**Keywords:** Food protein-induced enterocolitis syndrome, Down syndrome, Oral food challenge test

## Abstract

Down syndrome (DS) is a risk factor for severe food protein-induced enterocolitis syndrome (FPIES), with DS patients tending to have multiple-food FPIES. This is the first case where a DS infant with a history of severe chronic FPIES to milk and soy could effectively be introduced with some untested high-risk foods through hospital-based oral food challenges (OFCs).

The infant is a 20-month-old girl with DS, who was diagnosed with milk- and soy-induced FPIES. Considering her history of intensive care unit care for severe FPIES reactions, we considered that introducing other high-risk foods, such as wheat and hen’s egg (white and yolk), at home was not appropriate for her. We offered hospital-based OFCs effectively and safely by introducing wheat and hen’s egg as high-risk foods against FPIES to the 20-month-old infant. As a result, she tolerated soy-based seasoning, wheat, and egg whites without any symptoms, but she developed frequent vomiting after ingesting egg yolk. We did a prompt intervention with intravenous fluid replacement to prevent severe adverse conditions. After discharge, she exhibited an FPIES symptom as a consequence of ingesting green peas and miso; hence, we recommended the elimination of peas, in addition to soy, milk, and egg yolk, from her diet. She remained symptom-free since adhering to this dietary regimen.

In severe FPIES children, it is encouraged to introduce unconsumed high-risk foods in the hospital safely to avoid severe reactions at home and prevent unnecessary food eliminations.

## Introduction

Several recent reports have demonstrated a high frequency of severe and multiple-food protein-induced enterocolitis syndrome (FPIES) in children with Down syndrome (DS) ^(1), (2)^. Children with DS often suffer from poor weight gain due to the avoidance of certain foods. Additionally, there are no established guidelines for introducing untested high-risk foods to populations at high risk for FPIES. This case study presents an infant with DS who has a history of severe multiple FPIES to milk and soy, who was successfully introduced with high-risk foods ^(3)^, including wheat and egg white, through hospital-based oral food challenges (OFCs). The study confirmed an FPIES reaction to egg yolk. This is the first report demonstrating the effective and safe introduction of high-risk foods in a hospital setting to children with DS and severe FPIES.

## Case Report

**Figure 1.**
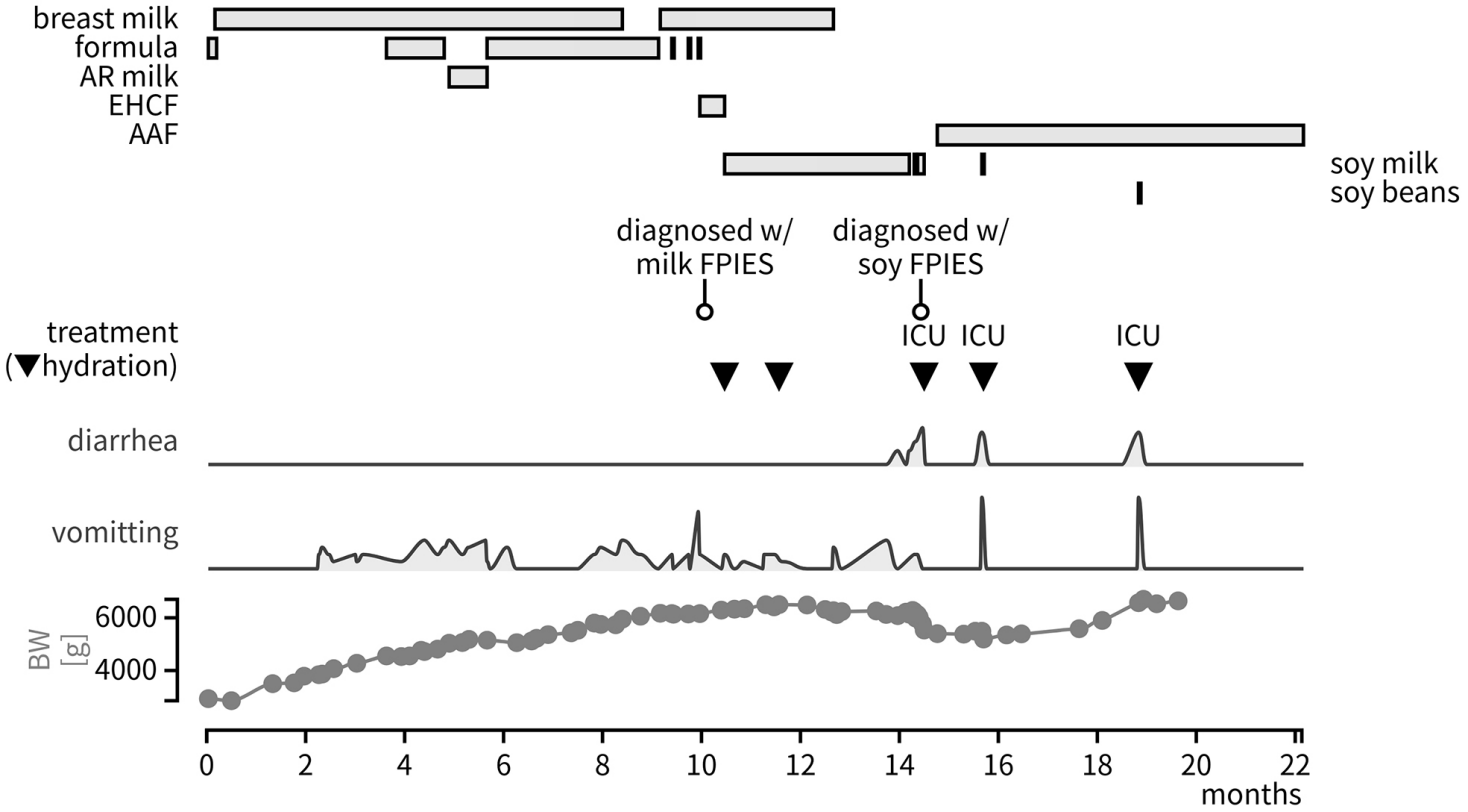
Patient’s past clinical course AR milk: anti-regurgitation milk; EHCF: extensively hydrolyzed casein-based formula; AAF: amino acid-based formula; and BW: body weight.

The infant was a girl with DS. She was born at 37 weeks of gestation and has a history of Tetralogy of Fallot, but no gastrointestinal surgeries. Frequent vomiting appeared 3 h post-ingestion of infant formula, leading to a diagnosis of chronic milk-induced FPIES at 9 months of age. The formula was switched to soy milk, after which, she experienced the same symptom at 13 months of age. This suggested complications of chronic soy-induced FPIES (Figure 1). ICU admission was required several times due to hypovolemic shock when she accidentally ingested soy products.

**Figure 2.**
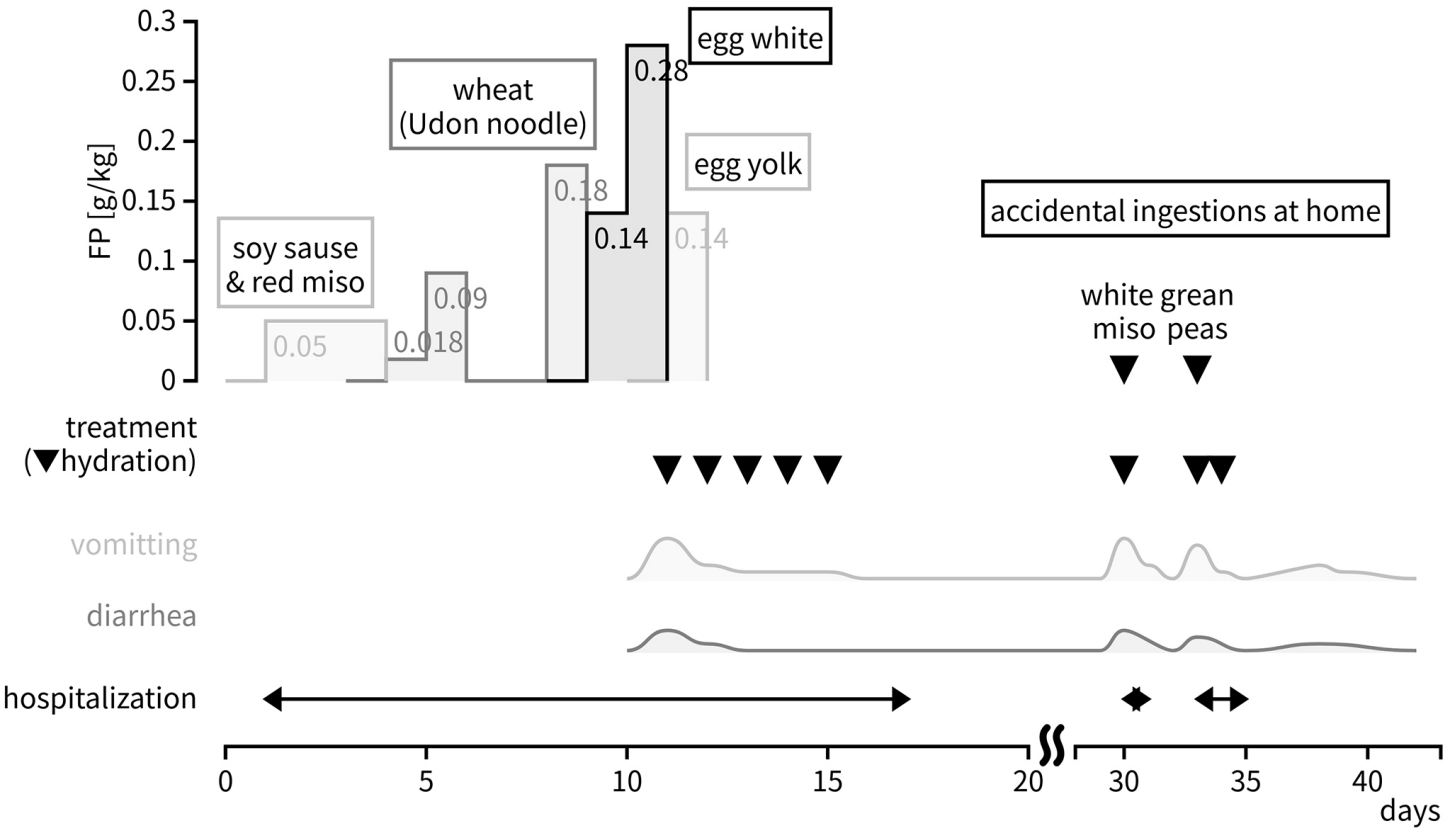
Patient’s clinical course after admission After discharge, she was readmitted twice to our hospital with the FPIES symptom following the accidental ingestion of green peas in a packaged food and miso soup. Moreover, although she was allowed to have miso due to the OFC results, the difference in the protein content between white miso and red miso triggered the symptoms. FP: food protein weight per body weight.

At 20 months of age, she was referred to our allergy center for further assessment and treatment. At that time, she was already tolerant of fish, meat, rice, vegetables, and fruits. Given her history of severe multiple FPIES to milk and soy, we conducted hospital-based OFCs (Figure 2) to introduce her to other high-risk foods available in our country, such as wheat and hen’s egg ^(3)^. Our OFC protocol followed international consensus guidelines for FPIES. The details of which can be found in the Supplementary Material. She tolerated soy-based seasonings (i.e., soy sauce and red miso), Udon noodles (i.e., wheat), and boiled egg whites without symptoms, but she developed frequent vomiting after taking 5 g of egg yolk (0.12 g of protein/body weight). Prompt intervention with intravenous fluid replacement was done; hence, we were able to prevent severe adverse conditions.

After discharge, she was readmitted to our hospital twice due to FPIES symptoms following accidental ingestion of green peas from packaged food and miso soup. Although OFCs indicated tolerance to miso, the protein content variation between white and red miso subsequently triggered the symptoms.

We provided the patient’s family with additional instructions as regards dietary elimination before discharge. She has remained symptom-free since adhering to this dietary regimen.

## Discussion

This case highlights the effectiveness and the safety of hospital-based OFCs for the introduction of untested high-risk foods in children with history of severe FPIES, particularly in children with DS.

We documented herein a successful case of OFCs implemented on a patient with a history of severe FPIES. Although it is recommended to introduce high-risk foods in a hospital setting ^(3)^, no definite consensus exists as regards tailoring the approach to individual risks. A previous study showed that all children with DS and FPIES demonstrate severe reactions ^(2)^. DS is considered a risk factor for severe and multiple FPIES ^(1)^ because of the high comorbidity of surgical gastrointestinal disorders ^(2), (4)^ and their unique immune system profiles ^(5)^. The trisomy of chromosome 21 induces interferonopathy, leading to increased TNF-α levels ^(6)^, which consequently leads to the influx of antigens into the intestinal submucosa ^(7)^. Complications, such as cardiovascular diseases, may also exacerbate general conditions when symptoms occur. In this case, OFCs were effectively and safely conducted following our protocol, without the need for ICU care. Introducing high-risk foods to high-risk patients through hospital-based OFCs with safer protocols should be highly recommended to avoid unnecessary food avoidance.

In this case, we observed FPIES symptoms induced by both soy and green peas, as well as soy-based seasoning. Recently, there has been an increase in legume allergies partly caused by the rising popularity of legumes in “healthy diets.” Although cases of FPIES to legumes other than soybeans or peanuts exist, few has reported multiple FPIES to soybeans and peas. The cross-reactivity between soy and other peas in immediate-type food allergy is thought to be uncommon ^(8)^. However, patients with a history of severe soy FPIES and their caregivers should be aware of the potential risks of cross-reactivity with other peas. In soy FPIES, soy-based seasonings, such as soy sauce or miso, are often tolerated. However, this infant exhibited a positive reaction to white miso at home despite having no previous reaction to red miso in the hospital. Red miso undergoes a longer fermentation process compared to white miso. Longer fermentation results in a greater breakdown of soy proteins ^(9)^, which reduces the reaction risk. In addition to this, 12.5% of patients with FPIES may show FPIES symptoms to foods, which were negative in previous OFCs ^(10)^; hence, we should be aware of the risks. These are the main reasons for readmission after OFCs in this patient. This case highlighted the importance of considering the specific details of what the patients ingest, even the soy-based seasoning. In conclusion, high-risk FPIES children are encouraged to be introduced with high-risk foods in a hospital setting to avoid severe reactions at home. All healthcare professionals should be aware that promptly referring to an allergy specialist is necessary in case patients with food allergies have unnecessary food eliminations.

## Article Information

### Conflicts of Interest

None

### Author Contributions

CJ and KY established the concept of this case study. All authors followed the case in the hospital. KH, DS, TY, MO, DH, KU, SH, FI, SH, and KT are in charge of outpatient care and inpatient OFC. TF and YO supervised this case. CJ wrote the first draft of the manuscript. All authors critically reviewed the manuscript and approved the final version.

### Approval by Institutional Review Board (IRB)

Not applicable. Informed consent was obtained from the patient’s parents.

## Supplement

Supplementary Material
