# Supplementary material for: Hospital-based Introduction of Untested High-risk Foods for Down Syndrome Infant with Severe Food Protein-induced Enterocolitis Syndrome: A Case Report
Source: JMA J. 2024 Dec 6;8(1):306–9. doi: 10.31662/jmaj.2024-0188 (PMC11799443; doi:10.31662/jmaj.2024-0188)
Supplement: Supplementary Material [file 2433-3298-8-1-0306-s001.pdf]

Supplementary information: OFC Protocol in our hospital setting

OFCs are conducted for three purposes: diagnoses/ remission/ confirmation of the safety of untested food

As suggested in international consensus guidelines[1], the target challenge dose is 0.06-0.6 g protein per kg body weight (kg BW). The dose can be adjusted according to the patient's age or severity. The target protein loading dose is low (0.06 g protein/kg BW) if the previous symptom with the target food is severe, and a standard dose (0.3 g protein/kg BW) is mild to moderate. A peripheral venous line is prepared before OFC if there is a risk of severe symptoms.

The OFC is conducted with 1/50th of the target dose, and the dose increases in the following order: 1/50th, 1/10th, 1/2, and then a full dose. Up-dosing was performed only once per day. The full challenge dose is not to exceed 3 g protein or 10 g whole food.

The protocol shown above shall not be applied to a case with untested food. Depending on each risk of the target food, the dose can be adjusted.

Ex. For a 10-kg infant

Food	FPIES severity	Loading Food Dose to a 10 kg infant				Target protein dose per BW
		1st day 1/50	2nd day 1/10	3rd day 1/2	4th day full	
Egg Yolk	Severe	0.06 g	0.3 g	1.5 g	3 g	0.06 g/kg
	Mild to moderate	0.3 g	1.6 g	8 g	16 g	0.3 g/kg
Egg White	Severe	0.12 g	0.6 g	3 g	6 g	0.06 g/kg
	Mild to moderate	0.6 g	3 g	15 g	30 g	0.3 g/kg
Lactose-free milk	Severe	0.7 ml	3.5 ml	17 ml	35 ml	0.06 g/kg
	Mild to moderate	3.5 ml	18 ml	88 ml	177 ml	0.3 g/kg
Soy (Tofu)	Severe	0.2 g	1.2 g	6 g	12 g	0.06 g/kg
	Mild to moderate	1.2 g	6 g	30 g	60 g	0.3 g/kg
Rice	Severe	0.5 g	2.4 g	12 g	24 g	0.06 g/kg
	Mild to moderate	2.4 g	12 g	60 g	120 g	0.3 g/kg

Ex. in this case : 7-kg infant/ OFC was conducted for untested foods

- Egg Yolk (20 g protein/100 g): 5 g - 10 g (0.14 g - 0.28 g protein/kg BW)
- Egg White (10 g protein/100 g): 10 g - 20 g (0.14g - 0.28 g protein/kg BW)
- Udon noodles (2.6 g protein/100 g): 5 g – 25 g – 50 g (0.018 g - 0.09 g - 0.18 g protein/kg BW)
- Red miso (11 g protein/100 g): 3g (0.05 g protein/kg BW)
- Soy sauce (6.7 g protein/100g) : 5g (0.05 g protein/kg BW)

References:

1. Nowak-Węgrzyn A, Chehade M, Groetch ME, et al. international consensus guidelines for the diagnosis and management of food protein-induced enterocolitis syndrome: Executive Summary-Workgroup Report of the Adverse Reactions to Foods Committee, American Academy of Allergy, Asthma & Immunology. J Allergy Clin Immunol. 2017 Apr;139(4):1111-26.
2. Nishimura K, Yamamoto-Hanada K, Sato M, et al. Remission of Acute Food Protein-Induced Enterocolitis Syndrome Confirmed by Oral Food Challenges in Japan. Nutrients. 2022 Oct 7;14(19):4158.
